# QTc interval prolongation in critically ill patients: Prevalence, risk factors and associated medications

**DOI:** 10.1371/journal.pone.0199028

**Published:** 2018-06-13

**Authors:** Flávia Medeiros Fernandes, Eliane Pereira Silva, Rand Randall Martins, Antonio Gouveia Oliveira

**Affiliations:** 1 Integrated Multiprofessional Health Residency Program—Adult Intensive Care Unit, Pharmacy Department, Health Sciences Centre, Universidade Federal do Rio Grande do Norte, Natal, RN, Brazil; 2 University Hospital Onofre Lopes, Health Sciences Centre, Universidade Federal do Rio Grande Norte, Natal, RN, Brazil; 3 Pharmacy Department, Health Sciences Centre, Universidade Federal do Rio Grande do Norte, Natal, RN, Brazil; University of Bern, University Hospital Bern, SWITZERLAND

## Abstract

**Purpose:**

To investigate the prevalence and risk factors of acquired long QT syndrome (LQTS) on admission to a general Intensive Care Unit (ICU), and to assess the risk of LQTS associated with prescribed medications.

**Methods:**

Prospective observational, cross-sectional study approved by the Institutional Review Board. Between May 2014 and July 2016, 412 patients >18 years-old consecutively admitted to the ICU of a university hospital were included. LQTS was defined as a QT interval on the admission electrocardiogram corrected using Bazett’s formula (QTc) >460 ms for men and >470 ms for women. All medications administered within 24 hours before admission were recorded. Logistic regression was used.

**Results:**

LQTS prevalence was 27.9%. In LQTS patients, 70.4% had ≥ 1 LQTS-inducing drug prescribed in the 24 hours prior to ICU admission versus 70.4% in non-LQTS patients (p = 0.99). Bradycardia and Charlson morbidity index score are independent risk factors for LQTS. Haloperidol (OR 4.416), amiodarone (OR 2.509) and furosemide (OR 1.895) were associated with LQTS, as well as another drug not yet described, namely clopidogrel (OR 2.241).

**Conclusions:**

The LQTS is highly prevalent in critically ill patients, ICU patients are often admitted with LQTS-inducing medications, and patients with slow heart rate or with high Charlson comorbidity index should be evaluated for LQTS.

## Introduction

The QT-interval, the length of time between the beginning of the QRS complex and the end of the T-wave of the electrocardiogram (ECG), is a marker of the duration of ventricular repolarisation. Its prolongation is associated with Torsade de Pointes (TdP), a rare polymorphic ventricular tachycardia. Although asymptomatic in most cases, TdP may present with palpitations, syncope, dizziness, convulsions, ventricular tachycardia, and sudden death [1]. This arrhythmia is the consequence of a longer duration of action potential in most ventricular myocardial cells, caused by a reduction of external repolarising currents and/or an increase of internal currents [2].

QT-interval prolongation, known as the long QT syndrome (LQTS), may have a congenital or an acquired aetiology. Several mutations have been described that cause changes in sodium and potassium ionic currents, leading to a prolonged action potential and to the LQTS [3]. Acquired causes include electrolyte abnormalities, sinus node dysfunction, high-grade atrioventricular block, myocardial ischemia and injury, and medication use [4]. The latter was the most common reason for QT-interval monitoring (57%) in a study performed in an Intensive Care Unit (ICU) [5].

Drug-induced LQTS has been, for decades, one of the most common causes for withdrawal of drugs from the market [6]. As of March 2013, 205 new drugs were evaluated for QT-interval prolongation, 46 of which were identified as being associated with the LQTS and five were denied registration [7].

Critically ill patients are at increased risk of developing LQTS because they frequently receive pro-arrhythmic drugs, in addition to commonly having other risk factors for TdP, such as renal or hepatic impairment, electrolyte disturbances and bradyarrhythmias [8]. In a study conducted in an Intensive Care Unit (ICU), approximately 69% of critically ill patients had at least one of the three American Heart Association criteria for QT-interval monitoring: use of drugs that are known to cause QT-interval prolongation, cardiac arrhythmias causing severe bradycardia, and hypokalemia or hypomagnesaemia [5].

Prospective studies in ICUs have shown prevalences of LQTS of 24% [5], 46.6% [9], 27.9% [10], 39% [11], 61% [12] and 52% [13]. Although LQTS is a frequent finding in critically ill patients and administration of QT-prolonging medications in the ICU setting is common, only a small number of prospective studies aimed at the accurate characterization of this problem have been published. In the last 10 years, only 10 studies on acquired LQTS in intensive care are found in the literature. Those studies were often based on retrospective data or had small sample sizes. Many of the published studies on QT interval prolongation are reviews or case reports. Studies that have focused on drug-induced LQTS commonly have rather simple statistical analyses, as they disregard the influence of other risk factors and study only the medications already reported in the literature as being associated with LQTS, without considering other drugs.

Therefore, we conducted a prospective study aimed at the investigation of the prevalence and risk factors for acquired LQTS in patients admitted to a general ICU and an assessment of the risk of LQTS associated with the prescribed medications.

## Material and methods

### Study design

From May 2014 to July 2016 we conducted an observational, cross-sectional study with prospectively collected data, in adult patients admitted to a general ICU in a university hospital. This project was approved by the Research Ethics Committee of University Hospital Onofre Lopes with number 666.969 and all study patients, or their legal representatives, signed an Informed Consent Form. During the study period, all patients of both genders, over 18 years-old, admitted to the ICU were invited to the study. Exclusion criteria were: patients with congenital LQTS, with complete left bundle branch block or other cardiac conduction defect disallowing QT-interval measurement, with an implanted pacemaker, who were admitted for less than 48 hours for routine monitoring after a diagnostic or therapeutic procedure, or who were admitted after elective surgery.

All patients had an ECG performed upon admission to the ICU and the QT-interval was measured manually by the same cardiologist throughout the study. The formula used to compute the corrected QT-interval (QTc) was Bazett’s formula [14]. For the measurement of the QT interval, a 12-lead ECG with a 10 second rhythm strip in DII was obtained with a CardioCare 2000 Bionet (Macrosul, Curitiba, Brazil) electrocardiograph, and the median length of QT intervals in six consecutive QRS complexes was determined. QTc prolongation was defined as a value greater than 460 ms for men and 470 ms for women [1]. Demographic and clinical data was collected from all patients, including cause of admission, co-morbidities, vital signs, blood biochemistry, arterial gasometry, Glasgow [15], SAPS II [16] and SOFA scores [17], and the Charlson Comorbidity Index [18]. All medications administered to the patient in the 24 hours prior to ICU admission were recorded.

### Statistical analysis

For the calculation of sample size, it was assumed that approximately half the patients admitted to the ICU would have LQTS, and that at least 5% would be prescribed a given LQTS-inducing drug. A sample size of 420 patients would afford 70% power for the identification of drugs associated with LQTS with a relative risk of 2.5 or greater. This sample size would allow the estimation of the prevalence of LQTS in ICU patients with a maximum error of ± 4.8 percentage points and estimations in the population with LQTS of relative frequencies with a maximum error of 6.8 percentage points.

Patient characteristics are summarized as absolute and relative frequencies or mean ± standard deviation. Population proportions are presented with 95% exact binomial confidence intervals (CI). The chi-square test and the Student’s t-test were used for comparisons of proportions and means, respectively. For the identification of risk factors for LQTS, patient variables were compared between LQTS and normal QTc patients and those variables with a p-value<0.10 were included into a logistic regression model for multivariate analysis. For the determination of the risk of LQTS, and of the average increase in QTc duration associated with each drug, we used multiple logistic regression and multiple linear regression, respectively, with adjustment by the SAPS II score, the Charlson comorbidity index, and the set of known risk factors for LQTS (age, sex, heart failure, hypokalemia and hypomagnesaemia). Results of multivariate analyses are presented as odds-ratios (O.R.) and 95% CI. All tests are two-sided. The significance level was set at the 5% level. No corrections for multiple comparisons were done. Stata 11 (Stata Corporation, Collegue Station, TX, USA) was used for the statistical analysis.

## Results

**Fig 1:**
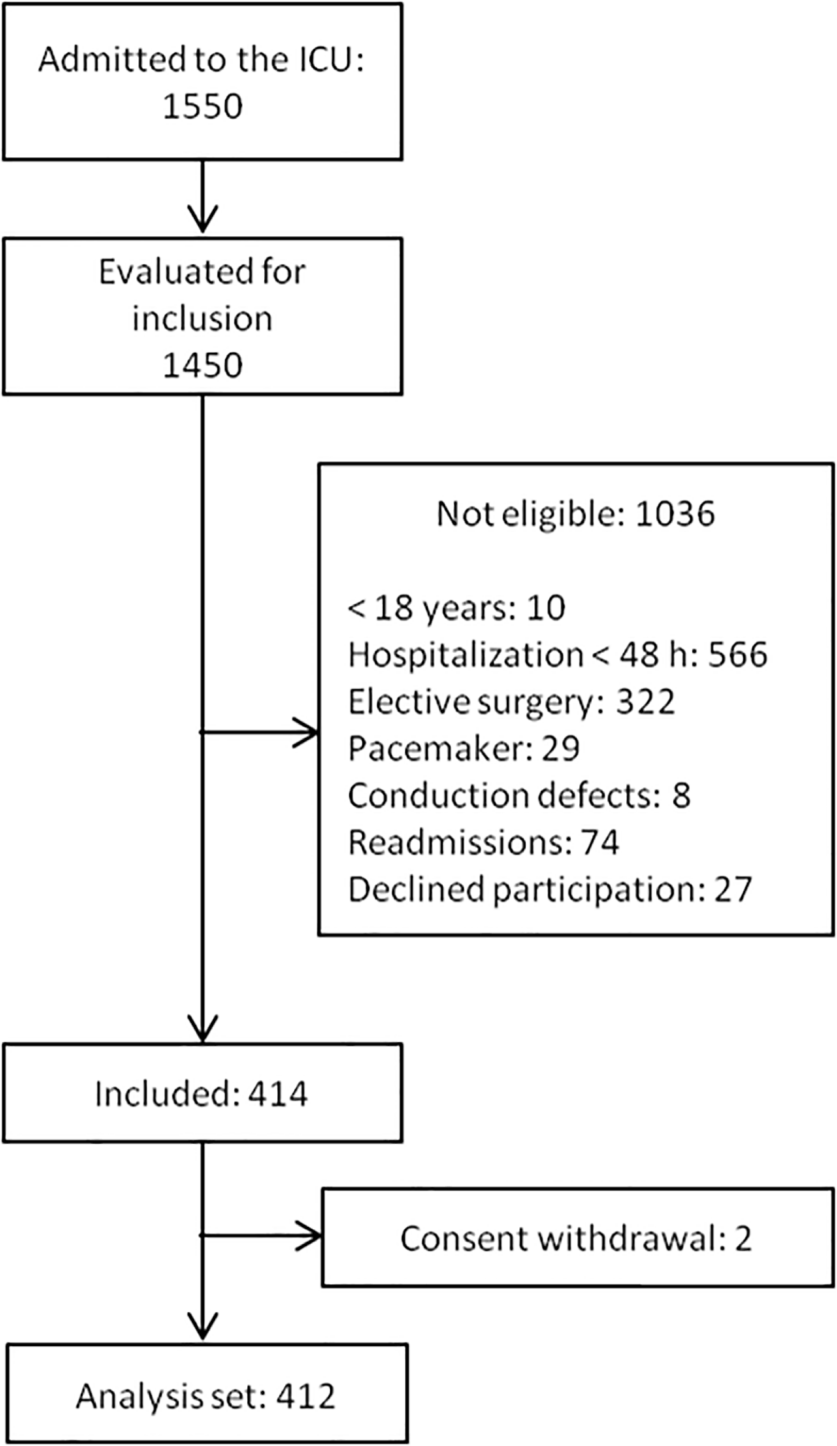
Patient accountability.

During the data collection period, 1550 patients were admitted to the ICU and 1450 (93.5%) were evaluated for inclusion. From these, 1009 patients were not eligible because of exclusion criteria, 27 patients refused participation and 2 patients withdrew consent (Fig 1). The final analysis set consisted of 412 patients with a mean age of 57.4 ± 16.1 years-old, 46.4% of whom were females. In 106 patients (25.7%) ICU admission followed emergency surgery. The most frequent admission diagnoses were myocardial infarction (136 patients, 33.0%), diabetes (125, 30.3%) and kidney disease (89, 21.6%), congestive heart failure (69, 16.8%), cancer (46, 11.2%), cerebrovascular disease (33, 8.0%) and liver disease (31, 7.5%). The mean SOFA score was 7.23 ± 4.13, the mean SAPS II score was 40.0 ± 19.1 and the mean Charlson comorbidity index was 3.57 ± 2.32. ICU mortality in this patient population was 17.7%. The characteristics of the population are shown in Table 1.

**Table 1:** Characteristics of the study population.

Patient Characteristics		
Female sex (n, %)	191	46.4
Age, years	57.4	16.1
Type of admission (n, %)		
Medical	306	74.3
Emergency surgery	106	25.7
Mechanical ventilation (n, %)	104	25.2
Charlson comorbidity index	3.57	2.33
Charlson probability of death, %	58.7	35.8
SOFA score	7.23	4.14
SAPS II score	40.0	19.1
SAPS II probability of death	30.5	29.8
Glasgow score	5.87	9.74

Values are mean and standard deviation unless otherwise specified.

**Fig 2:**
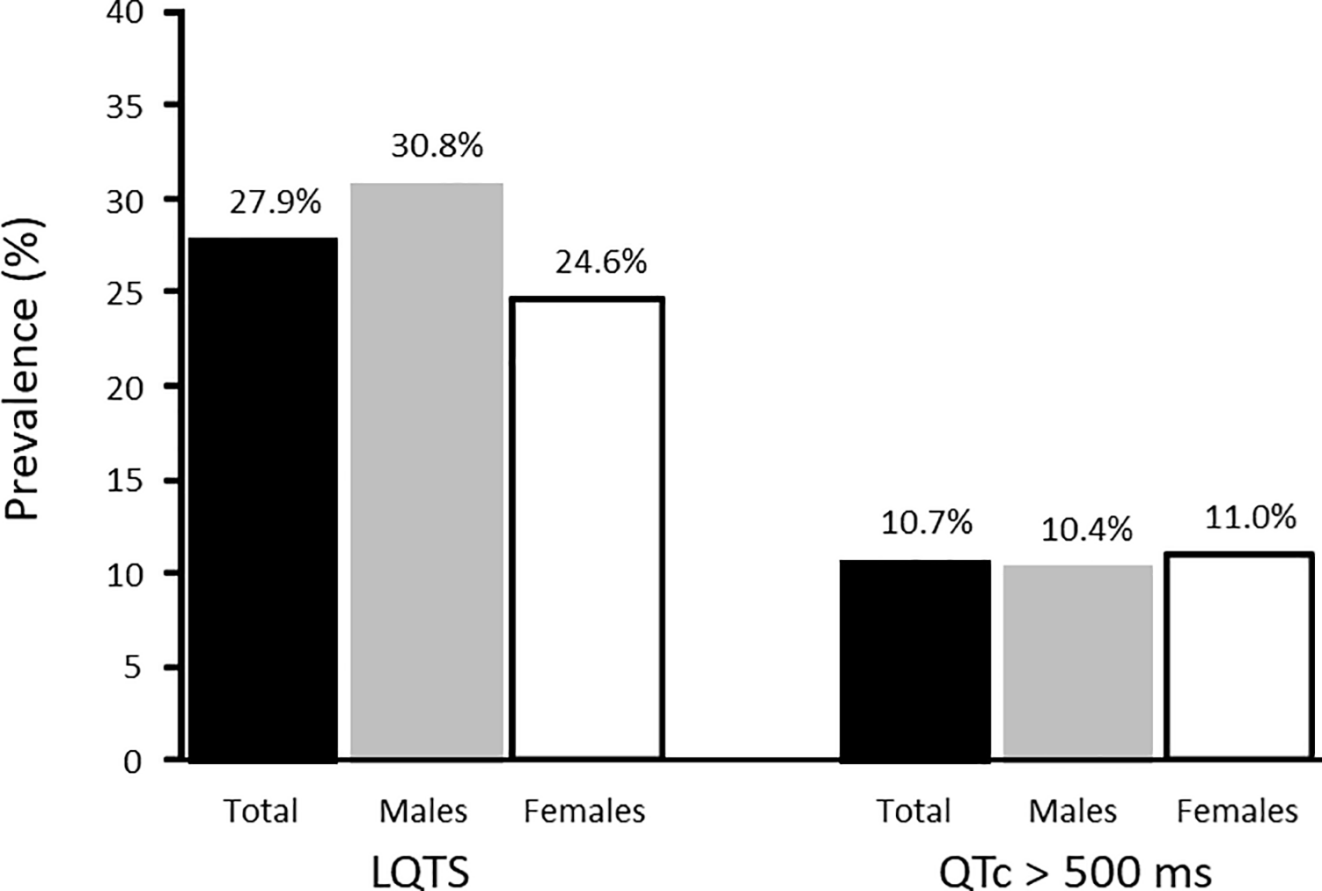
Sex-specific prevalence rates of LQTS among patients admitted to an Intensive Care Unit.

The prevalence of LQTS at ICU admission was 115/412 patients (68 males, 47 females), with corresponding population estimates of the prevalence rate of 27.9% (95% CI 23.6%– 32.5%), 30.8% (95% CI 24.8%– 37.3%) among males and 24.6% (95% CI 18.7%– 31.3%) among females (p = 0.16) (Fig 2). The prevalence rate of QTc >500 ms was 10.7% (95% CI 7.9% - 14.1%), 10.4% (95% CI 6.7% - 15.2%) in males and 11.0% (95% 6.9% - 16.3%) in females. The mean QTc value among LQTS patients was 508 ± 59.1 ms (range 461–877 ms), 505 ± 64.5 ms in males and 512 ± 50.4 ms in females (p = 0.48). In non-LQTS patients, the mean QTc value was 420 ± 29.5 ms.

**Fig 3:**
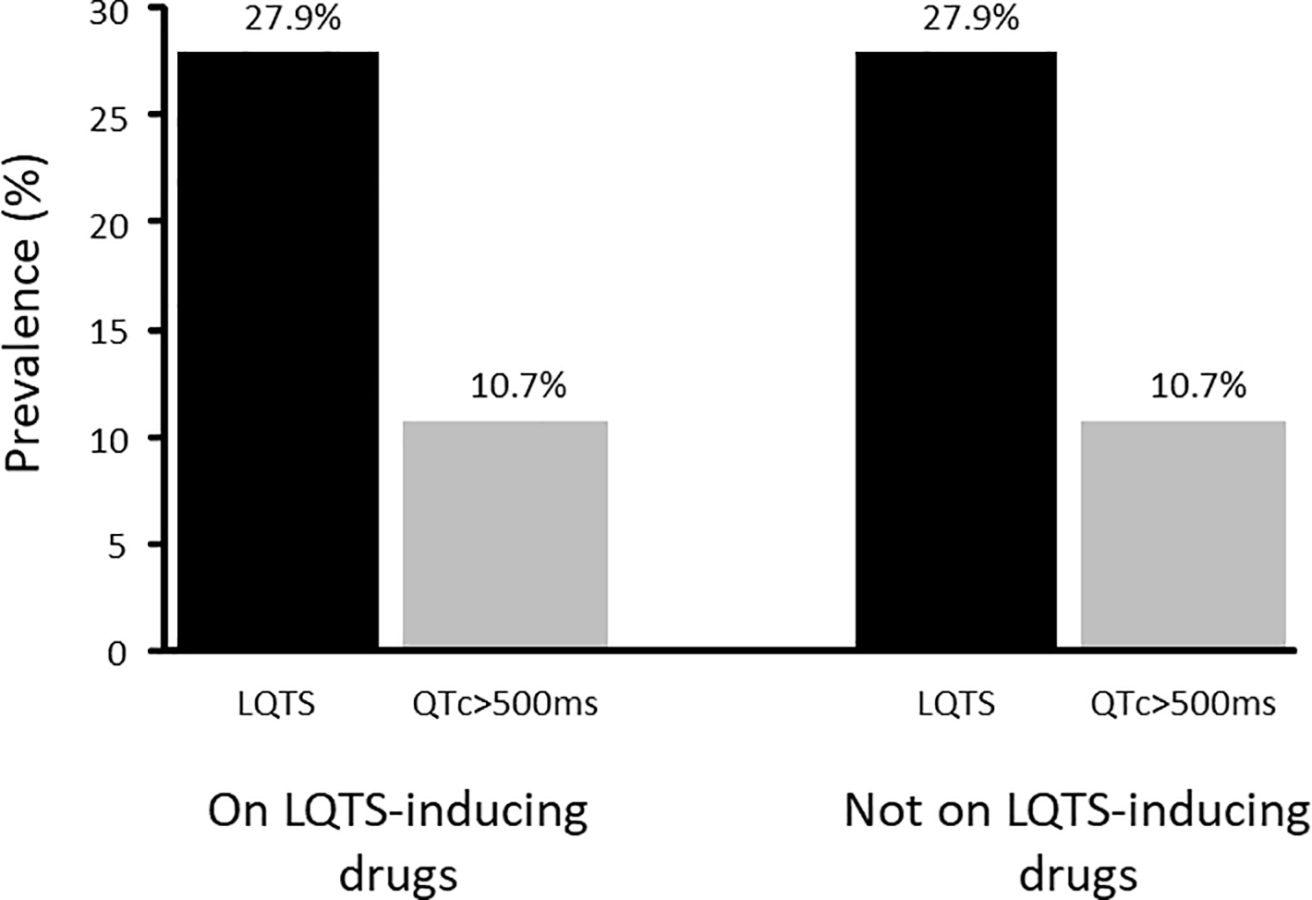
Prevalence rates of LQTS among users and non-users of QT-interval prolonging drugs in the 24 hours prior to ICU admission.

At the time of admission, 290 patients (70.4%, 95% CI 66.0%– 74.8%) had a prescription of at least one drug known to be associated with LQTS. Among LQTS patients, 81/115 patients (70.4%, 95% CI 61.2%– 78.6%) were receiving a LQTS-inducing drug, while 209/297 patients with normal QTc (70.4%, 95% CI 64.8%– 75.5%) were medicated with one of those drugs (p = 0.99). In the group of 290 patients prescribed with a LQTS-inducing drug, the prevalence of LQTS is 27.9% (95% CI 22.8%– 33.5%) and the prevalence of QTc>500 ms is 10.7% (95% CI 7.4%– 14.8%). In the 122 patients not prescribed with those drugs, the prevalences are, respectively, 27.9% (95% CI 20.1%– 36.7%) and 10.7% (95% CI 5.8–17.5%) (Fig 3). The differences are not statistically significant.

Compared to patients with normal QTc, patients with LQTS on ICU admission are older, have slower heart rate, lower body temperature, greater Charlson comorbidity index, and are more likely of having an ICU admission because of myocardial infarction, heart failure or diabetes (Table 2). However, multivariate analysis revealed that the risk of LQTS on ICU admission was independently associated only with a slower heart rate (O.R. 0.986, 95% CI 0.976–0.996, p = 0.005) and with the Charlson morbidity index score (O.R. 1.157, 95% CI 1.053–1.271, p = 0.002).

**Table 2:** Patient characteristics of the groups with normal QTc and with LQTS.

Patient characteristics	Normal QTc	LQTS	p
Age (years)	55.8 ± 16.6	61.3 ± 14.1	0.002
Female sex	144 (48.5%)	47 (40.9%)	0.16
Emergency surgery	75 (25.3%)	31 (27.0%)	0.72
Admission Diagnosis			
Myocardial infarction	87 (29.3%)	49 (42.6%)	0.01
Heart failure	42 (14.1%)	27 (23.5%)	0.02
Diabetes	80 (26.9%)	45 (39.1%)	0.02
Nephropathy	65 (21.9%)	24 (20.9%)	0.82
Systolic blood pressure (mmHg)	128.8 ± 32.6	125.5 ± 30.1	0.34
Diastolic blood pressure (mmHg)	73.0 ± 18.9	72.9 ± 17.8	0.97
Mean blood pressure (mmHg)	91.5 ± 22.4	90.4 ± 21.1	0.66
Heart rate (bpm)	93.2 ± 23.0	86.2 ± 21.2	0.005
Body temperature (C)	35.8 ± 0.98	35.6 ± 0.94	0.01
Blood chemistry			
Sodium (mmol/L)	137.8 ± 6.97	138.1 ± 6.68	0.70
Potassium(mmol/L)	4.51 ± 0.95	4.43 ± 0.94	0.43
Bicarbonate (mmol/L)	22.5 ± 7.33	22.1 ± 6.50	0.57
Calcium (mg/dL)	8.88 ± 1.12	8.79 ± 1.28	0.53
Magnesium (mg/dL)	2.03 ± 0.43	2.10 ± 0.49	0.21
Urea (mg/dL)	77.7 ± 62.7	73.5 ± 52.7	0.52
Creatinine (mg/dL)	1.87 ± 2.09	1.69 ± 1.79	0.43
Mechanical ventilation	69 (23.2%)	35 (30.4%)	0.13
PaO2 (mmHg)	115 ± 53.4	120 ± 60.7	0.61
SAPS II score	40.2 ± 19.7	39.6 ± 17.4	0.82
Charlson comorbidity index	3.36 ± 2.25	4.12 ± 2.44	0.003

Values are mean ± standard deviation or number (percentage).

In order to assess the risk of LQTS for each drug known to prolong the QT-interval, we performed a series of logistic regression analyses to obtain estimates of the odds ratio of LQTS associated with each drug. The results are presented in Table 3. The drugs that seem to present greater risk of LQTS are haloperidol (O.R. 4.416), amiodarone (O.R. 2.509) and furosemide (OR 1.895).

**Table 3:** Risk of LQTS associated with drugs known to induce LQTS.

LQTS-inducing medicines	n	Risk of LQTS				QTc increase (ms)		
		Adjusted OR	95% CI		p	Mean	95%CI	
Furosemide	76	1.895	1.035	3.471	0.04	13. 0	-0.01	26.1
Haloperidol	10	4.416	0.982	19.862	0.05	19.4	-13.9	52.6
Amiodarone	20	2.315	0.828	6.475	0.11	22.0	+0.1	44.9
Metronidazole	19	1.746	0.545	5.600	0.35	1.98	-22.1	26.0
Fluconazole	12	1.727	0.453	6.577	0.42	8.69	-19.9	37.3

Odds-ratios and mean increases adjusted for risk factors of LQTS (age, sex, hypokalemia, hypomagnesaemia, heart failure), SAPS II score and Charlson comorbidity index.

The same analysis was done for all other 263 different drugs that patients were medicated with at the time of ICU admission. Clopidogrel (O.R. 2.000, 95% CI 1.039–3.850, p = 0.04) was the only drug showing statistically significant association with LQTS after adjustment by risk factors for LQTS and measures of disease severity (SAPS II and Charlson comorbidity index). Clopidogrel was being administered to 97 patients at ICU admission and their average QTc interval was 452 ± 52.7 ms, range 344–626 ms.

## Discussion

The main results of our study were that over one quarter of patients admitted to a general ICU present LQTS, that the presence of LQTS is strongly associated with the use of LQTS-inducing medicines already described in the literature, such as haloperidol, amiodarone and furosemide, but also with a drug not yet described, namely clopidogrel, and that a slower heart rate and a greater Charlson comorbidity index score on ICU admission are independent risk factors for LQTS.

QT prolongation is the main cause of drug-induced malignant arrhythmias. It is associated not only with the properties or doses of drugs, but also with the presence of risk factors such as age, gender, drug interaction, genetic predisposition, electrolyte changes or comorbidities [19]. The main mechanism of action involved is blockade of the rapid component of the potassium current, which prolongs the period of ventricular repolarisation. The delay in myocardial repolarisation increases the propensity for the development of early depolarisations, which are manifested in the electrocardiogram as ventricular ectopy, associated with the development of TdP, ventricular fibrillation and sudden death [1].

QTc interval values up to 440 ms are considered normal. From 440 to 460 ms and from 440 to 470 ms are considered borderline in men and women, respectively. Above these values the QT-interval is considered prolonged [1]. However, arrhythmias are more frequently associated with values above 500 ms [20].

There are several formulas used to correct QT interval according to heart rate (Bazzet, Fridericia, Framingham, Hodges, Nomogram and Rautaharju), and there is still no consensus on the best formula to be used. A study concluded that Bazzet’s formula should be avoided when the heart rate differs from 60 bpm, with the Hodges and Nomogram formula being more adequate in assessing the difference between baseline QTc and during antiarrhythmic therapy [21]. Another study also observed that Bazzet’s formula overestimated the number of patients with prolonged QTc interval, while the Fridericia and Framingham formulas showed the best correction rate and significantly improved the prediction of mortality at 30 days and 1 year [14]. Despite this, Bazzet's formula continues to be widely used both in clinical practice and for research purposes [10, 12, 22–25], and so it was chosen as the QT interval correction formula in this study.

The overall incidence of drug-induced TdP is difficult to estimate, due to its transient nature and the need for electrocardiographic evaluation for correct detection. A study in Germany estimated an incidence of 0.26 cases per million inhabitants [26]. A retrospective study analyzed ICU admissions due to drug-induced malignant arrhythmias for 6 years and estimated an annual incidence of 0.1% (33 cases out of 5788 admissions), in which TdP occurred in 55% of cases (18/33) [22].

Despite the low prevalence of TdP, LQTS has been seen quite frequently in ICUs. Kozik et al retrospectively analysed continuous ECG recordings of 88 patients during the first 72 hours of ICU admission and detected QTc interval> 500 ms in 46% (40/88) [23], while Ridruejo et al searched the hospital database of a medical ICU of 17 beds for a period of 7 years and identified only 88 cases of LQTS (> 450 ms for men and> 460 ms for women), among 9730 patients (0.9%) [24]. A retrospective study performed at an emergency department of a tertiary university for 3 months identified a prevalence of 35% (544/1558, 95% CI 32–37%), in which 8% had QTc greater than 500 ms (120/1558, 95% CI of 6 to 9%) [27], a result similar to that observed in another retrospective study, in which 38% (5650/14804) presented QTc> 450 ms for men and greater than 460 ms for women and 12% (1711/14804) had QTc> 500 ms [25].

A prospective study that included only ICU patients using LQTS-associated medications, detected a prevalence of 61% (121/200) of QTc prolongation (greater than 470 ms in women and greater than 450 ms in men), and of 40% (79/200) of QTc> 500 ms [12]. Tisdale et al, in a prospective one year study in 2 intensive care units, detected a prevalence of 27.9% (251/900) of QTc> 470 ms in men and QTc> 480 ms in women, where 18.2% (164/900) had QTc> 500 ms (10). Another study observed only critical post-surgical patients for 15 months and identified a prevalence of 67% (172/257) of QTc prolongation > 440 ms [28].

A single centre study performed at an ICU for 2 months using continuous QT interval monitoring for 15 minutes detected a prevalence of 52% (26/50) of QTc> 500 ms [13], while a multicenter study of 6 ICUs over 2 months, also using continuous QT monitoring, found a prevalence of 24% (252/1039) of QTc> 500 ms [8].

The prevalence found in our study of 27.9% (115/412) of QTc greater than 460 ms in men and greater than 470 ms in women, and 10.7% of QTc> 500 ms refers only to the moment of ICU admission and must not be confused with the prevalence during the period of ICU stay. We have found a high prevalence of LQTS, with no significant difference between genders, and that over 70.4% of LQTS patients were prescribed with LQTS-inducing drugs at admission on the ICU. Other studies have identified percentages of LQTS-inducing drug use of 45% (6661/14804) [25] and 34.7% (87/251) [29].

In this study, it was observed that the prevalence of LQTS is the same whether patients did or did not use LQTS-inducing medications before ICU admission. Therefore, we believe that all patients admitted to the ICU should be evaluated for the presence of LQTS, regardless of whether they are using LQTS-inducing drugs.

In our study, age was associated with the acquired LQTS, which has been described as an independent risk factor by several reviews [1, 30, 31]. Astrom et al. found age 65 and older as the second highest risk factor (72%) in cases of TdP reported in the pharmacovigilance system in Sweden between 1991 and 2006 [7]. The drug utilization profile of the aged population (including antidepressants, psychotropic, antiarrhythmics, among other drugs), together with the concomitant use of several drugs, which increases the risk of drug interactions causing QTc prolongation and TdP, as well as the presence of other risk factors, may explain why this age group is more susceptible [9]. Many studies point to the female gender as an important risk factor for QTc prolongation, reporting a two- to three-fold increase in the risk of TdP [8], possibly through reduction of oestrogen-mediated repolarisation [32]. However, our data did not evidence a gender association with QTc duration in ICU patients.

Slow heart rate, hypothermia, high Charlson score, myocardial infarction, heart failure and diabetes were more prevalent in the LQTS group. However, in multivariate analysis only slow heart rate and high Charlson score were identified as risk factors for QTc prolongation at ICU admission, as has also been reported in other studies [33, 34].

Class IA, IC and III antiarrhythmics are the drugs most commonly involved in drug-induced TdP cases. Amiodarone was the drug most associated with cases of TdP (113 cases/222 non-cases) reported to the FDA Adverse Event Reporting System (AERS) for 4 years (2004 to 2007) [35]. However, a large number of non-cardiac medications have also been described as inducers of QT prolongation and TdP, and although they are less risky, they are widely used in clinical practice and therefore can reach a large number of people [36]. Among these drugs are antimicrobials, antipsychotics, antidepressants, antiemetics, immunosuppressants, anaesthetics, muscle relaxants, diuretics, anticonvulsants, opioids, as well as a number of others [37].

A few studies have looked into the risk of QTc prolongation associated with several drugs, but have analysed associations only with univariate models, without considering the influence of other risk factors [13, 24]. In our study, multivariate analysis adjusted by a set of confounding factors estimated the odds-ratios of LQTS for drugs known to be associated with QT prolongation. Only a recent study estimated for furosemide the risk of 3.26 (95% CI 1.41 to 7.54) for QTc> 500 ms [38].

Only one recent study evaluated the effect of commonly used medications over the duration of the QTc-interval. In a sample of 1270 hospitalized patients, clopidogrel was found to be associated with a risk of QTc> 450/470 ms of 2.49 (95% CI 1.21 to 5.10, p< 0.05), and enoxaparin with a risk of QTc prolongation> 30 ms of 1.47 (95% CI 0.96 to 2.24), although without statistical difference [38]. Our study also found that patients taking clopidogrel had increased QTc interval.

An implication for clinical practice, given the high prevalence of the LQTS at ICU admission and the frequent prescription of LQTS-inducing drugs in critically ill patients, is that clinical pharmacists should maintain a high level of alertness in the evaluation of the pharmacotherapy of ICU patients with LQTS, looking for electrolytic disturbances, identifying important drug interactions, evaluating the use of LQTS-inducing drugs in severely ill cardiac patients, and adjusting dosage schedules in renal failure patients, among other possible actions [33].

One limitation of our study is that it was conducted at a single ICU, which may diminish the generalisability of the results. However, we believe that the kind of pathology, the severity of the clinical conditions, and the clinical practice in our unit is fairly similar to the majority of ICUs in university hospitals. Another limitation is that the QT-interval was measured on a single ECG strip and, because of the known variability of the QT-interval, we may have missed a few cases of LQTS. On the other hand, the QT-interval was measured manually, which is more accurate than automated measurements, and always by the same observer, which eliminated inter-observer variability. Our results are only for LQTS present on ICU admission and we did not evaluate the prevalence throughout the entire ICU stay.

## Conclusions

In conclusion, our study showed a high prevalence of LQTS in critically ill patients, that those patients often are admitted with LQTS-inducing medications, and that patients with slow heart rate or with high Charlson comorbidity index should be evaluated for LQTS.

## Supporting information

S1 File Dataset. (XLSX)
